# Partial Oxygen Pressure Affects the Expression of Prognostic Biomarkers HIF-1 Alpha, Ki67, and CK20 in the Microenvironment of Colorectal Cancer Tissue

**DOI:** 10.1155/2016/1204715

**Published:** 2016-11-15

**Authors:** Lirong Zhang, Yu Hu, Ning Xi, Jie Song, Wenjing Huang, Shanshan Song, Yiting Liu, Xianying Liu, Yingjun Xie

**Affiliations:** ^1^Department of Pathology, China-Japan Union Hospital of Jilin University, Changchun 130031, China; ^2^Hospital Office, People's Liberation Army No. 93246, Changchun 130051, China; ^3^Department of Gynecology and Obstetrics, The Second Affiliated Hospital of Jilin University, Changchun 130041, China; ^4^Jilin Province Yitong Secondary Health Vocational School, Siping 130000, China; ^5^Department of Radiation, School of Public Health, Jilin University, Jilin 130031, China; ^6^Department of Hepatobiliary Pancreatic Surgery, The Second Affiliated Hospital of Jilin University, Changchun 130041, China

## Abstract

Hypoxia is prognostically important in colorectal cancer (CRC) therapy. Partial oxygen pressure (pO_2_) is an important parameter of hypoxia. The correlation between pO_2_ levels and expression levels of prognostic biomarkers was measured in CRC tissues. Human CRC tissues were collected and pO_2_ levels were measured by OxyLite. Three methods for tissue fixation were compared, including formalin, Finefix, and Finefix-plus-microwave. Immunohistochemistry (IHC) staining was conducted by using the avidin-biotin complex technique for detecting the antibodies to hypoxia inducible factor-1 (HIF-1) alpha, cytokeratin 20 (CK20), and cell proliferation factor Ki67. The levels of pO_2_ were negatively associated with the size of CRC tissues. Finefix-plus-microwave fixation has the potential to replace formalin. Additionally, microwave treatment improved Finefix performance in tissue fixation and protein preservation. The percentage of positive cells and gray values of HIF-1 alpha, CK20, and Ki67 were associated with CRC development (*P* < 0.05). The levels of pO_2_ were positively related with the gray values of Ki67 and negatively related with the values of HIF-1 alpha and CK20 (*P* < 0.05). Thus, the levels of microenvironmental pO_2_ affect the expression of predictive biomarkers HIF-1 alpha, CK20, and Ki67 in the development of CRC tissues.

## 1. Introduction

Colorectal cancer (CRC) is the most common malignancy and third-leading cause of cancer death worldwide [1]. With the development of biotechnology, the changes for some functional proteins have been revealed in CRC tissues [2–4]. Hypoxia is prognostically important in cancer therapy and it has a deep impact on malignant development of various cancers [5, 6]. HIF-1 (hypoxia inducible factor-1), a kind of hydrocarbon nuclear translators, plays an important role in oxygen homeostasis [7] and consists of two subunits, HIF-1 alpha and HIF-1 beta. HIF-1 alpha is oxygen-mediated factor, which affects HIF bioactivity. HIF-1 alpha can activate genes and their translated proteins, such as erythropoietin [8], glucose transporters [9], and vascular endothelial growth factor (VEGF) [10]. The change for HIF-1 alpha has been detected in colorectal cancer (CRC) [11, 12] and several authors have described its importance in angiogenesis and CRC growth [13, 14]. Ki67, a nuclear protein, affects cellular proliferation and it reflects a reversible change in cellular bioactivity during neoplastic progression [15]. Ki67 has been demonstrated to be involved in cancer invasion and angiogenesis and it may be an important CRC biomarker [16]. A statistically significant relationship has been found between HIF and Ki67 [17] while Ki67 is also an important biomarker for CRC diagnosis [16, 18]. Thus, hypoxia may affect the expression of Ki67. Cytokeratin 20 (CK20) is a well-known biomarker for detecting circulating tumor cells (CTCs) in the patients with metastatic CRC [19].

Oxygen deficiency is a critical issue in cancer development [20]. To confirm tumor hypoxia, partial oxygen pressure (pO_2_) is an important factor of physiological state, and it indicates the balance between oxygen delivery and consumption [21]. Also, it can be measured in cancer tissues. The study of different-sized tumors showed that pO_2_ levels decreased as tumor volume increased [22], suggesting that there is a negative relation between pO_2_ levels and cancer development. The OxyLite system provides a rapid method to determine the levels of pO_2_ in tumors [22]. HIF is a key protein responsible for the cellular adaptation to low levels of oxygen [23]. HIF is activated as a result of a drop in pO_2_ [24]. Cytokeratin (CK) is a marker for epithelial cells [25, 26]. The intestinal epithelial cells play an important critical role in the immune response within the intestinal tissue and they are involved in the responses to hypoxic situation [27]. Previous studies showed that the expression of cytokeratin was enhanced in the cells with low oxygen tension [28]. The proliferative cell marker Ki67 is induced under hypoxia conditions in which Ki67 has been found to be highly expressed [17, 29, 30]. Therefore, the expression of HIF-1 alpha, CK20, and Ki67 may be affected by pO_2_ levels in the microenvironment of tissues from CRC.

Tissue fixation and staining is a basic method in the research and diagnosis of functional proteins in CRC tissues [31]. Formalin is one of the most widely used chemical reagents for tissue fixation. The fixative is toxic and can be a threat to public health. Furthermore, the detection of low abundance proteins is extremely difficult because of long-time fixation resulting in the degradation of low abundance proteins. To overcome the problem, Finefix, alcohol-based fixative, is a good solution for tissues fixation [32]. A microwave approach to normal fixation reduces much considerably time to perform it over conventional methods. To avoid important protein degradation, microwave heating may be a potential method for speeding up tissue penetration and fixation. Previous reports showed that microwave tissue processing shortened processing times without side effects on tissue morphology and microwave stabilization significantly improved detection of important proteins in frozen sections [33]. To optimize the fixation procedure, microwave irradiation can be helpful because it can reduce the fixative time by heating tissue inside. Present work was performed to assess the effects of pO_2_ on the expression of predictive biomarkers in the recurrence of CRC tissues fixed by Finefix with the help of microwave step.

## 2. Materials and Methods

### 2.1. Participants

All the protocols and informed consent forms were approved by the Human Research Ethic Committee from China-Japan Union Hospital of Jilin University (Changchun, China). Additionally, all the data were obtained under a certificate of confidentiality obtained from the same organization. The diagnostic standards for CRC were used according to previously reported [34].

### 2.2. Tissue Specimen Collection and pO_2_ Measurements

From May 17, 2011, to May 16, 2013, 234 patients were examined and 168 patients were diagnosed with CRC and confirmed by pathologists. Sinker-assisted endoscopic submucosal dissection was effective for the removal of CRC [35]. The data for tumor size were collected from colonoscopy. pO_2_ was measured immediately by using OxyLite Systems (OxyLite 4000, Oxford Optronix, Oxford, UK) in different tumors at 37°C. A total of 168 cancerous colorectal tissues were collected from different patients and pO_2_ was measured at China-Japan Union Hospital of Jilin University after CRC determination.

### 2.3. Tissue Fixation

CRC tissues were routinely fixed with 10% neutral-buffered formalin, paraffin-embedded, and dehydrated according to standard protocols. Fixation time was 24 h. Some tissue samples were cut into 2 mm pieces and fixed in Finefix (Milestone Srl, Sorisole, Torre Boldone, Italy) as quickly as possible according to manufacturer's instructions. For all tissues, the time from tissue isolation to immersion in Finefix method was less than half an hour. Some Finefix treated issues were placed into cassettes and treated with Thermo Scientific™ Tissue Wave™ 2 Microwave Processor (Thermo Fisher Scientific (China) Co., Ltd., Beijing, China) at 150 W for 60 min and at 650 W for 15 min. Two-gram tissue samples were placed in 50 mL tubes with 20-mL Finefix. The tubes floated on their sides in the container were thermoelectrically cooled. In this case, no agitation of Finefix occurred during microwave irradiating. To maintain the temperatures no more than 30°C in the inside of tubes at 150 W, the temperature was set at 20°C. The temperature was automatically reset to 10°C at 650 W. Subsequently, the samples were dehydrated and paraffin-embedded according to the manufacturer's recommendations.

### 2.4. Hematoxylin and Eosin (H&E) Stain

Hematoxylin and Eosin (H&E) stain was performed according to a previous report [36].

### 2.5. Immunohistochemistry (IHC) Staining

IHC staining was conducted by using avidin-biotin complex technique for detecting the antibodies to cytokeratin 20 (CK20), carcinoembryonic antigen (HIF-1 alpha), and cell proliferation factor Ki67. Slides were treated with 2% H_2_O_2_ in methanol for 1 h to inactivate endogenous peroxidase. The sections were washed twice with PBS and then incubated with blocking serum for 1 h. This was followed by incubation with rabbit anti-human CK20 antibody (Cat. number 119-15444), or HIF-1 alpha antibody (Cat. number 119-12223) and/or Ki67 antibody (Cat. number 119-15444) from RayBiotech, Inc. (Norcross, GA, USA) at room temperature in a wet chamber. Then sections were washed with PBS and incubated with biotinylated goat anti-rabbit secondary antibody (Cat. number sc-2040, Santa Cruz Biotechnology, Inc., Santa Cruz, CA, USA) for 1 h. Sections were then treated with ABC solution (Vector Laboratories, Burlingame, CA, USA) for 1 h, washed with PBS, and incubated with DAB for 10 min. Counterstaining experiment was performed with Harris hematoxylin (Sigma-Aldrich; St Louis, MO, USA). Percentage of positive cells (0%–100%) was calculated with a cut-off value of 1% of positively stained cells.

### 2.6. IHC Imaging

Immunostains were reordered by using an Olympus IX73 inverted microscope (Olympus (China) Co., Ltd., Shanghai, China). Image analysis software was used to control image acquisition and image processing. Five images were obtained from different locations in each slide. All images were quantitatively analyzed by using the software HPIAS-1000 (Wuhan Qianping Image Technology Co. Ltd. Wuhan, China). Grayscale color's value was measured to stand for the intensity of the immunostaining and the levels of functional proteins.

### 2.7. Statistical Analysis

Student's *t*-test was used to compare the gray values for the levels of HIF-1 alpha, CK20, and Ki67 from different samples. Spearman's rank correlation coefficient was used to measure the correlation between tumor size and the levels of pO_2_ and between pO_2_ levels and expression levels of the biomarkers. The *P* value was regarded as significant if it was less than 0.05.

## 3. Results

### 3.1. Baseline Characters of CRC Patients

A total of 168 patients were recruited according to CRC diagnosis criteria. The median follow-up was 18.7 months (ranging from 0.1 to 34.1 months). The mean age of the study sample was 63 ± 27 years. Most patients (68%) were in 48- to 70-year age range. Ninety-nine (58.9%) patients were male and 69 (41.1%) were females. LDH levels were higher in 112 CRC patients (66.7%). The metastatic sites often mostly occurred in liver, followed by lung, lymph nodes, and ascites. All the CRC patients were diagnosed by the same doctor. Of all patients, 27% (45 cases) of CRC were located in the proximal colon, 24% (40 cases) of CRC were located in the distal colon, and 49% (83) of CRC were located in the rectum. Among these cases, 21% (36 cases) of CRC tissues accounted for small size (less than 300 mm^3^), 36% (60 cases) for middle size (300–600 mm^3^), 28% (47 cases) for big-size (600–900 mm^3^), and 15% (25 cases) for super size.

### 3.2. pO_2_ in Different-Size CRC Tissues

**Figure 1:**
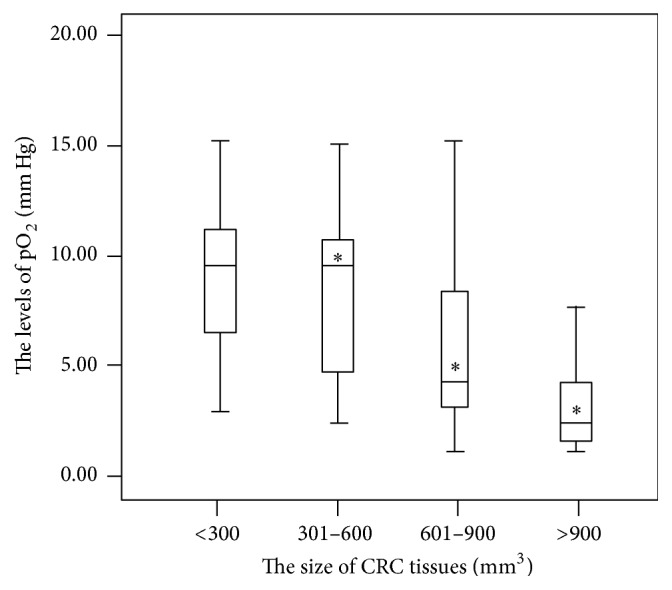
The levels of pO_2_ were negatively associated with the tissue size of CRC. ^*∗*^ *P* < 0.05 via a small-size CRC tissue (<300 mm^3^). The bars in the boxes were average values of pO_2_ levels and the boxes represented 90% of the samples. The error bars were above or below the boxes.

In small-size CRC tissues, pO_2_ levels were increased, and mean values were more than 10 mm Hg (Figure 1). The values were reduced with the increase of CRC tumor size. The mean levels of pO_2_ were less than 2.5 mm Hg in super-size CRC tissues (Figure 1). There was negative correlation between the tissue size of CRC and pO_2_ levels.

### 3.3. H&E Staining Analysis

**Figure 2:**
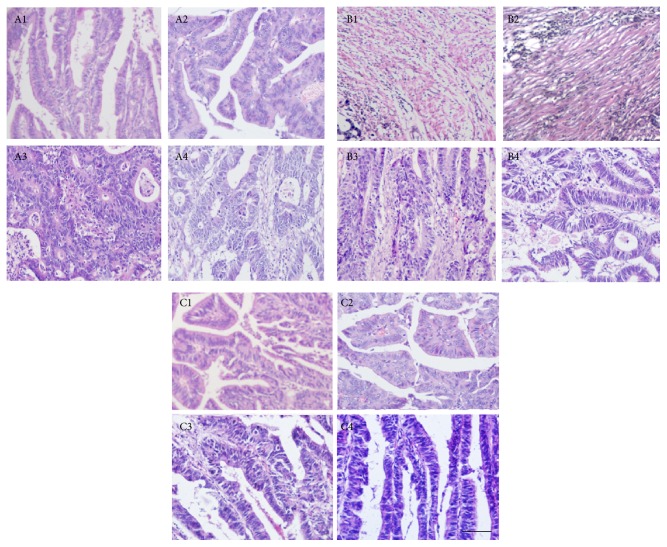
H&E staining analysis of different-size CRC tissues. The numbers 1, 2, 3, and 4 represent small-size CRC tissues (<300 mm^3^, *n* = 36), middle-size CRC tissues (301–600 mm^3^, *n* = 60), big-size CRC tissues (601–900 mm^3^, *n* = 47), and super-size CRC tissues (>900 mm^3^, *n* = 25) under 100x magnification, respectively. Scale bar: 50 *μ*m. The letters A, B, and C represent Finefix, formalin-fixed, and Finefix-plus-microwave-fixed tissues, respectively.

H&E sections were compared in different-size CRC tissues.  Figure 2 shows a typical image of H&E-stained CRC tissues. Formalin fixation and Finefix fixation were comparable (Figures 2(A) and 2(B)). Comparatively, Finefix-plus-microwave method showed clearer results. Dark blue nuclei were easily separated from cytoplasm in red in H&E staining. The staining pattern was maintained in all the tissues in MFG groups. H&E staining provided a clearer picture of overall morphology of CRC tissues. Furthermore, the nuclei were light blue and backgrounds were light red in small-size cancer (Figure 2(C)). Comparatively, the blue became thicker when the size of CRC tissues increased (Figures 2(C1)–2(C4)). The color depth was positively related with CRC development.

### 3.4. Percentage of Positive Cells

The percentage of positive cells from formalin- and Finefix-fixed tissues was lower than that from Finefix-plus-microwave fixed tissues for CK20 and Ki67 (Table 1). For HIF-1a, the percentage of positive cells of Finefix and Finefix-plus-microwave fixation was comparable and higher than formalin fixation. For Ki67, the percentage from formalin fixation was higher than that from Finefix fixation. In Finefix-plus-microwave-fixed tissues, the percentage of positive cells for HIF-1 alpha was from 50.4% ± 4.6% to 90.2% ± 5.7% with a cut-off value of 1% of positively stained cells (Table 1). Comparatively, the percentage of positive cells for CK20 was from 38.2% ± 3.2% to 60.6% ± 4.9% in the CRC from small size to super size. In contrast, the percentage of positive cells for Ki67 was from 79.8% ± 6.1% to 45.0% ± 3.4%. These results suggest that the percentage of positive cells for HIF-1 alpha and CK20 was positively related with CRC size while the percentage of positive cells for Ki67 was negatively related with CRC size.

### 3.5. Identification of HIF-1 Alpha in CRC Tissues

**Figure 3:**
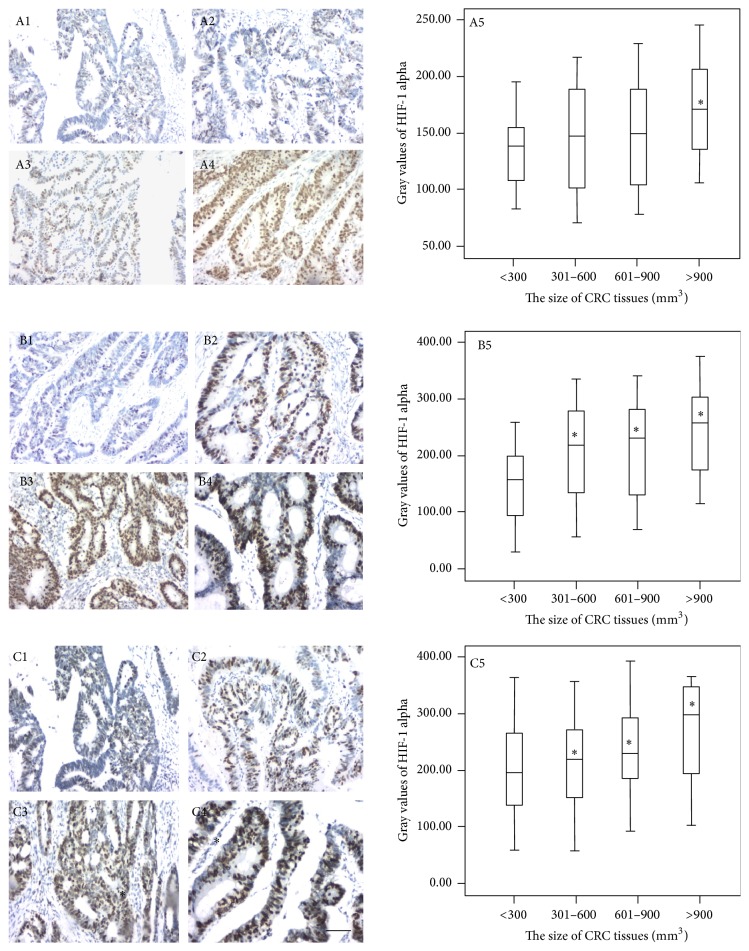
Immunohistochemical analysis for the expression levels of HIF-1 alpha in different-size CRC tissues. The numbers 1, 2, 3, 4, and 5 represent small-size CRC tissues (<300 mm^3^, *n* = 36), middle-size CRC tissues (301–600 mm^3^, *n* = 60), big-size CRC tissues (601–900 mm^3^, *n* = 47), and super-size CRC tissues (>900 mm^3^, *n* = 25) under 200x magnification and the gray values of HIF-1 alpha, respectively. The letters (A), (B), and (C) represent Finefix, formalin-fixed, and Finefix-plus-microwave-fixed tissues, respectively. ^*∗*^ *P* < 0.05 via a small-size CRC tissue. Scale bar: 50 *μ*m. The bars in the boxes were average activities and the boxes represented 90% of the samples. The error bars were above or below the boxes.

HIF-1 alpha is an important biomarker for CRC diagnosis and detected in CRC tissues [37]. DAB stain could be easily distinguishable from both HIF-1 alpha and the light hematoxylin intensity in the background, while maintaining the staining pattern in the tissue. HIF-1 alpha was positive brown stain while hematoxylin counterstain was blue. The stain intensity from formalin-fixed tissues was lower than that from Finefix- and Finefix-plus-microwave fixed tissues (Figure 3). The stain intensity of Finefix and Finefix-plus-microwave fixation was comparable (Figures 3(B) and 3(C)). In Finefix-plus-microwave-fixed tissues, Figures 3(C1)–3(C4) show a typical image of IHC-stained HIF-1 alpha in CRC tissues. The nuclei were brown from small-size cancer tissue and dark brown from middle- to super-size CRC tissues. The IHC staining showed similar results in all tissues. Figure 3(C5) shows that the gray values were significantly higher in super-size cancer tissues than in small size (*P* < 0.05). Furthermore, the color depth was positively related with CRC development. IHC staining provided a clearer picture of the overall morphology of CRC tissue. Variations in staining within the cells were evident, and stained nuclei were easily distinguished from low-intensity areas of IHC stain. Differentiation between cytoplasm and nuclei was improved.

### 3.6. Identification of CK20 in CRC Tissues

**Figure 4:**
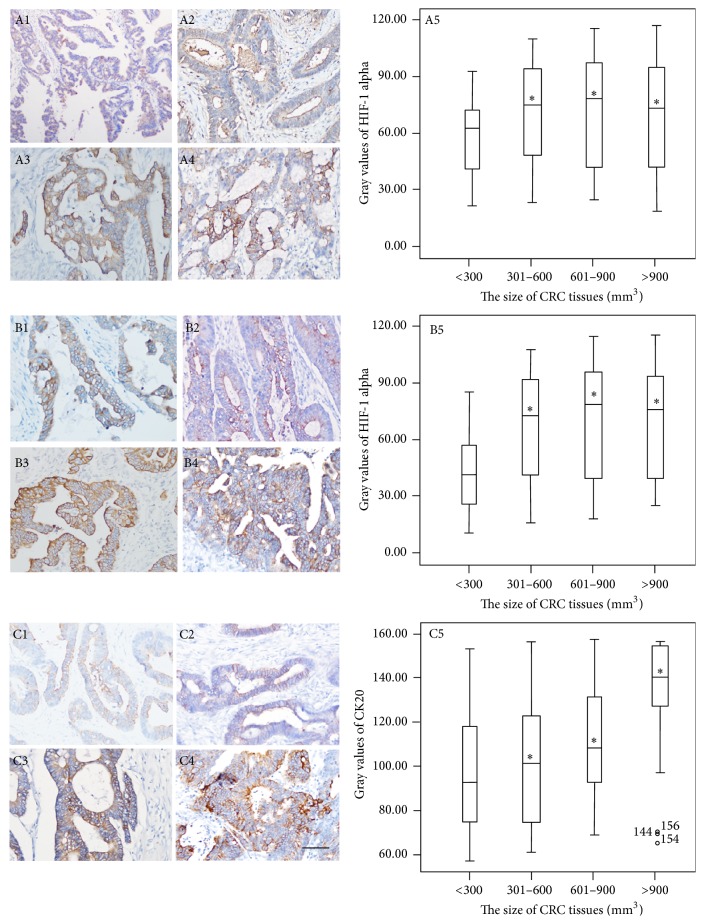
Immunohistochemical analysis for the expression levels of CK20 in different-size CRC tissues. The numbers 1, 2, 3, 4, and 5 represent small-size CRC tissues (<300 mm^3^, *n* = 36), middle-size CRC tissues (301–600 mm^3^, *n* = 60), big-size CRC tissues (601–900 mm^3^, *n* = 47), and super-size CRC tissues (>900 mm^3^, *n* = 25) under 200x magnification and the gray values of CK20, respectively. The letters (A), (B), and (C) represent Finefix, formalin-fixed, and Finefix-plus-microwave-fixed tissues, respectively. ^*∗*^ *P* < 0.05 via a small-size CRC tissue. Scale bar: 50 *μ*m. The bars in the boxes were average activities and the boxes represented 90% of the samples. The error bars were above or below the boxes.

CK20 is an important biomarker for CRC diagnosis and was detected in CRC tissues [38]. The stain intensity was comparable among three methods (*P* > 0.05) (Figure 4). In Finefix-plus-microwave-fixed tissues, Figures 4(C1)–4(C4) show a typical image of IHC-stained CK20 in CRC tissues. The cytomembrane was in brown in small-size cancer tissues and dark brown in middle-size to super-size CRC tissues. The IHC staining showed similar results in all tissues. Figure 4(C5) shows that the gray values were significantly higher in super-size CRC tissues than in small-size tissues (*P* < 0.05). Furthermore, the color was positively related with the development of CRC in both groups. The results indicated that microwave pretreatment has high sensitivity for detecting CRC biomarker CK20 during IHC analysis.

### 3.7. Identification of Ki67 in CRC Tissues

**Figure 5:**
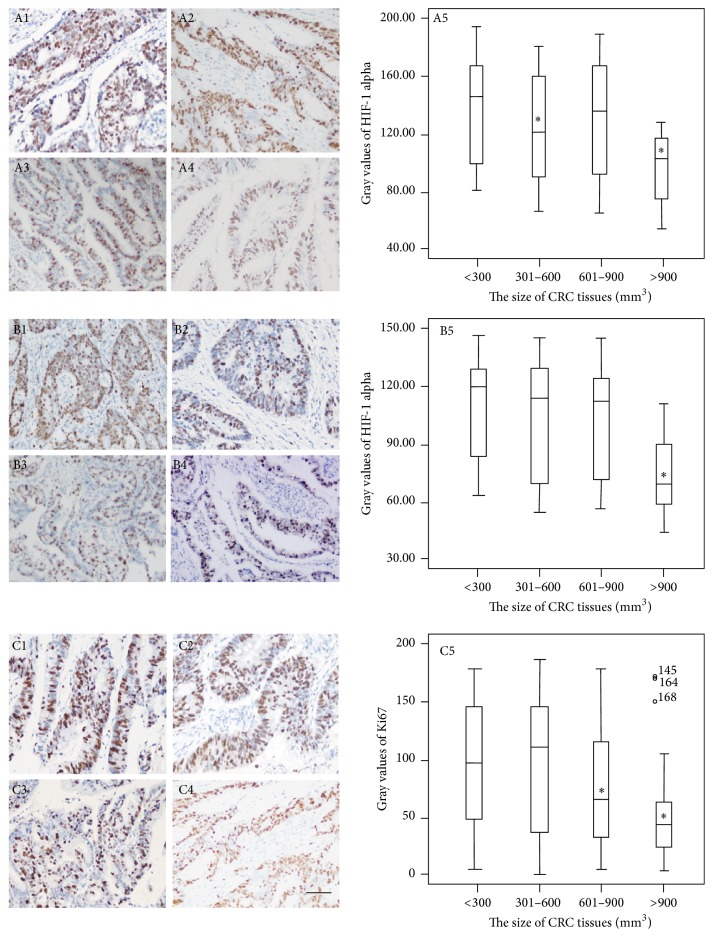
Immunohistochemical analysis for the expression levels of Ki67 in different-size CRC tissues. The numbers of 1, 2, 3, 4, and 5 represent small-size tissues (<300 mm^3^, *n* = 36), middle-size tissues (301–600 mm^3^, *n* = 60), big-size tissues (601–900 mm^3^, *n* = 47), and super-size tissues (>900 mm^3^, *n* = 25) under 200x magnification and the gray values of Ki67, respectively. The letters (A), (B), and (C) represent Finefix, formalin-fixed, and Finefix-plus-microwave-fixed tissues, respectively. ^*∗*^ *P* < 0.05 via a small-size CRC tissue. Scale bar: 50 *μ*m. The bars in the boxes were average activities and the boxes represented 90% of the samples. The error bars were above or below the boxes.

Ki67 also is an important biomarker for CRC diagnosis and was detected in CRC tissues [39]. The stain intensity from formalin-fixed tissues was higher than that from Finefix-fixed tissues while the stain intensity from formalin-fixed tissues was lower than that from Finefix-plus-microwave fixed tissues for Ki67 (Figure 5). In Finefix-plus-microwave-fixed tissues, Figures 5(C1)–5(C4) show a typical image of IHC-stained Ki67 in CRC tissues. The nuclei were dark brown from small-size to big-size cancer tissues and brown from super-size cancer tissues. Figure 5(C5) shows that the gray values were significantly higher in small-size than in super-size CRC tissues (*P* < 0.05). Furthermore, the color depth was negatively related with CRC development in all groups. The results indicated that microwave pretreatment and Finefix method has high sensitivity for detecting CRC biomarker Ki67 during IHC analysis.

### 3.8. The Stringent Relationship between the Levels of pO_2_ and the Size of CRC Tissues

**Figure 6:**
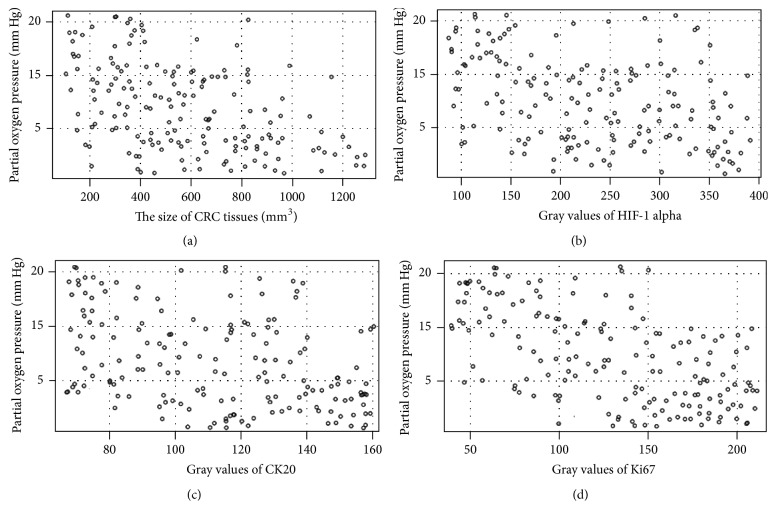
The association between pO_2_ levels and tissue size of CRC and/or protein levels of biomarkers in different-size CRC tissues. (a) The correlation between pO_2_ levels and tissue size of CRC. (b) The correlation between pO_2_ levels and the protein level of HIF-1 alpha in different-size CRC tissues. (c) The correlation between pO_2_ levels and the protein level of CK20 in different-size CRC tissues. (d) The correlation between pO_2_ levels and the protein level of Ki67 in different-size CRC tissues. Small-size CRC tissues (<300 mm^3^, *n* = 36); middle-size CRC tissues (301–600 mm^3^, *n* = 60); big-size CRC tissues (601–900 mm^3^, *n* = 47); super-size CRC tissues (>900 mm^3^, *n* = 25). Statistical values were measured by using a test for Spearman's rank correlation. If the values were between −1 and −0.5, there was a strong negative relation. If the values were between 0.5 and 1, there was a strong positive relation.

The results of Spearman's rank correlation coefficient indicated that the relationship between pO_2_ level and tissue size of CRC was strongly negatively related (Rho = 0.78, *P* < 0.05) (Figure 6(a)). The strong correlation between the levels of pO_2_ and the size of CRC tissues suggested that hypoxic microenvironment promotes CRC development. Hypoxia is a main factor for causing the risk of CRC.

### 3.9. The Stringent Relationship between the Levels of pO_2_ and the Expression Levels of HIF-1 Alpha, Ki67, and CK20 in CRC Tissues

The results of Spearman's rank correlation coefficient indicated that the relationship between pO_2_ levels and the protein levels of HIF-1 alpha and CK20 was strongly negatively related in CRC tissues (Rho = −0.72 and −0.69, resp., *P* < 0.05) (Figures 6(b) and 6(c)). In contrast, pO_2_ levels and the protein levels of Ki67 were strongly positively related in CRC tissues (Rho = 0.75, *P* < 0.05) (Figure 6(d)). The strong relation between the levels of pO_2_ and the size of CRC tissues suggested that hypoxic microenvironment promotes CRC development by affecting the protein expression of different biomarkers.

## 4. Discussion

The main aim of this study was to compare the levels of expression of Ki67, CK20, and Hif-1 alpha by an HIC approach according to specific pO_2_ determined in CRC samples. During the study, three fixative methods were compared: formalin, Finefix, and Finefix-plus-microwave. Among the three methods, the fixation time for formalin procedure is the longest. Additionally, most proteins cannot be preserved well after formalin or Finefix fixation. In contrast, the fixation time for Finefix-plus-microwave method is shorter and the proteins from samples can be preserved well. Finefix-plus-microwave fixation has the potential to replace formalin and improve Finefix performance in tissue fixation and protein preservation.

It is true that IHC on Finefix-fixed tissues requires optimization, but in most cases it requires recalibrating the antibodies because in comparison to formalin-fixed tissues the staining is enhanced as already reported [40]. For HIF-1 alpha, the stain intensity from formalin-fixed tissues was lower than that from Finefix- and Finefix-plus-microwave fixed tissues (Figure 3). The stain intensity of Finefix and Finefix-plus-microwave fixation was comparable (Figures 3(B) and 3(C)). For CK20, the stain intensity was comparable among three methods (*P* > 0.05) (Figure 4). For Ki67, the stain intensity from formalin-fixed tissues was higher than that from Finefix-fixed tissues while the stain intensity from formalin-fixed tissues was lower than that from Finefix-plus-microwave fixed tissues for Ki67 (Figure 5). Comparatively, microwave irradiation improves the penetration of fixatives and antibody solutions into CRC tissues, resulting in efficient fixation and reduction of nonspecific antigen [41]. After treatment with microwave heat and Finefix, the tissues have clear morphology with less tension and elasticity and harder nuclei [42]. The utilization of microwave offered a reproducible method for quantifying antigenicity.

HIF-1 alpha is an important biomarker for the hypoxic microenvironment of CRC, and hypoxic situation will activate the expression of HIF-1 alpha [14, 43–45]. Our results also suggest that hypoxic microenvironment with low pO_2_ increases the expression of HIF-1 alpha (Figure 6(b)). Significant changes were found not only in hypoxia related protein HIF-1 alpha, but also in cytoskeletal protein CK20 (Figure 6(c)). The role of Ki67 in the progression of CRC remains unclear. The combined expression of CD34 and Ki67 may be an important characteristic of CRC patients and can be used as predictive markers in the CRC development [16]. Present findings showed that the levels of Ki67 were negatively related with CRC development (Figure 6(d)). Especially in super-size CRC tissues, the levels of Ki67 reached the lowest level. Thus, Ki67 may be a dependent biomarker for CRC diagnosis, which was not consistent with previous reports [39, 46]. However, our results were consistent with a recent report [18], suggesting that other cellular mechanisms may be involved.

**Figure 7:**
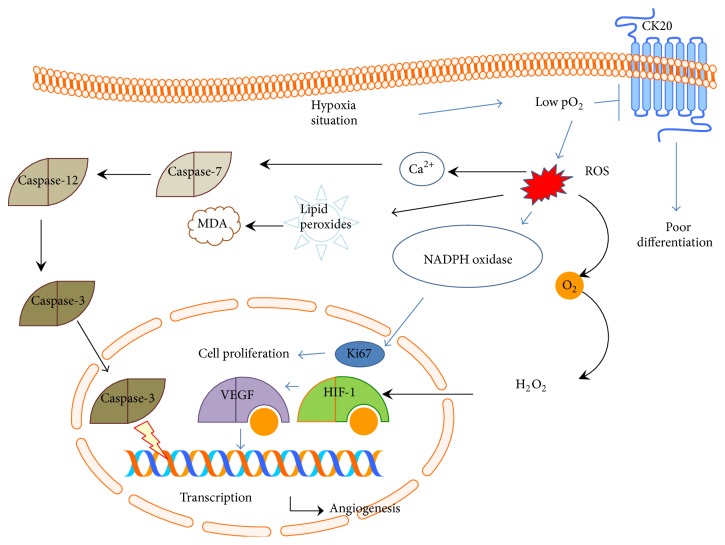
Low pO_2_ affects the activity of biomarkers of CRC. Low pO_2_ affects the activity of biomarkers of CRC via affecting ROS-mediated multiple pathways or CK20 pathway. The effects of these biomarkers lead to poor differentiation, cell proliferation, and angiogenesis.

The level of pO_2_ is negatively associated with the size of CRC tissues (Figure 6(a)) and the pO_2_ level provided a simple method to detect CRC development. Present findings showed that the levels of pO_2_ were negatively related with the progression of CRC, especially in super-size CRC tissues. On the other hand, the levels of pO_2_ were negatively related with levels of HIF-1 alpha and Ki67 but positively related with the levels of CK20. Previous study showed that the expression of cytokeratin was enhanced in the cells with low oxygen tension [10], suggesting that different CK may have different functions. In this sense, low pO_2_ could affect the activities of biomarkers of CRC. Low pO_2_ affects the activity of biomarkers of CRC via modifying ROS-mediated multiple pathways or CK20 pathway. The regulation of CK20 results in the cell poor differentiation. The changes of Ki67 and HIF-1 alpha will promote cell proliferation and angiogenesis (Figure 7).

There were following limitations for present work: (1) pO_2_ measurement may be unstable because of complex situations of CRC tissues. (2) This technique was not approved in other tissues, especially for the tissue with high brown pigmentation, which is most likely to be interpreted as positive as DAB stain. (3) Some proteins are only focally in benign space. (4) The biomarker-mediated metabolism was not analyzed here. For example, some nuclear proteins are only present in nuclei. The presence and distribution of stained nuclei will not be dependent on the fixing time or specific protocol. Therefore, further work is needed to provide statistical verification for the wide usage of microwave-treated Finefix.

Three functional proteins were detected in our current work. The tissue size of CRC is closely associated with pO_2_ levels, which is positively related with expression levels of Ki67 and negatively related with the levels of HIF-1 alpha and CK20, suggesting that partial oxygen pressure affects the expression of predictive biomarkers in the recurrence of colorectal cancer tissue fixed by Finefix. Additionally, there are the following advantages for microwave-stimulated Finefix fixation: (1) rapid, safe, and effective fixation; (2) well preservation of tissue morphology; (3) improvement of immune reactivity providing reliable and homogeneous immunostaining in cancer tissues; and (4) the reduction of protein degradation.

## Figures and Tables

**Table 1:** Percentage of positively stained cells.

Fixatives	Size of CRC	HIF-1 alpha	CK20	Ki67
Formalin	Small	38.6% ± 4.9%	33.7% ± 2.8%	59.4% ± 5.4%
	Middle	40.6% ± 5.7%	36.5% ± 3.2%	57.2% ± 4.2%
	Big	41.8% ± 6.3%	37.4% ± 3.6%	44.1% ± 4.5%
	Super	62.4% ± 6.8%	48.2% ± 4.1%	36.2% ± 3.7%
Finefix	Small	47.3% ± 3.5%	31.4% ± 2.5%	49.7% ± 4.8%
	Middle	64.9% ± 4.3%	34.8% ± 2.9%	47.4% ± 4.3%
	Big	66.2% ± 5.7%	38.5% ± 3.7%	38.1% ± 5.0%
	Super	85.1% ± 6.2%	49.2% ± 4.4%	31.5% ± 3.2%
Finefix plus microwave	Small	50.4% ± 4.6%	38.2% ± 3.2%	79.8% ± 6.1%
	Middle	65.7% ± 5.2%	47.2% ± 3.7%	68.2% ± 4.9%
	Big	70.3% ± 6.8%	51.5% ± 4.3%	52.3% ± 4.1%
	Super	90.2% ± 5.7%	60.6% ± 4.9%	45.0% ± 3.4%
